# ^15^O-water PET for evaluation of cardiopulmonary perfusion in complex cyanotic heart disease

**DOI:** 10.1186/s41824-020-0072-4

**Published:** 2020-02-13

**Authors:** S. Madsen, L. P. Tolbod, U. M. Mortensen, G. Andersen, K. Bouchelouche

**Affiliations:** 1 Department of Nuclear Medicine & PET Centre, Aarhus University Hospital, Palle Juul-Jensens Boulevard 99, DK-8200 Aarhus, Denmark; 2 Department of Cardiology, Aarhus University Hospital, Palle Juul-Jensens Boulevard 99, DK-8200 Aarhus, Denmark; 3 Department of Radiology, Aarhus University Hospital, Palle Juul-Jensens Boulevard 99, DK-8200 Aarhus, Denmark

**Keywords:** O-15-H2O PET, Water PET, ^15^O-water PET, Congenital heart disease, Lung perfusion, Myocardial blood flow

## Abstract

**Background:**

Dynamic ^15^O-water PET may provide information about cardiopulmonary circulation complementary to MRI and CT in complex cyanotic heart disease.

**Case presentation:**

We present a case in which a ^15^O-water PET scan was used for the first time to map the complex circulation in a univentricular heart patient with dual pulmonary blood supply. The pulmonary blood supply consisted of partially oxygenated blood led from the univentricle to the lungs by the pulmonary artery, plus of venous blood from the upper body lead by a bidirectional Glenn anastomosis to the right pulmonary artery. Despite the bidirectional Glenn anastomosis, the patient developed increasing cyanosis and was considered for heart transplantation. Pulmonary perfusion measurements using MRI were inconclusive due to metal artifacts, and the patient was referred for a ^15^O-water PET scan. The scan showed significant venovenous collaterals bypassing the lungs. Only the left upper lung lobe was properly perfused. The mean transit time from the superior vena cava to the left ventricle was approximately four times longer than would be expected from a healthy person.

**Conclusion:**

The case illustrates that ^15^O-water PET can complement CT and MRI for quantitative characterization of cardiopulmonary circulation in complex cyanotic heart disease.

## Background

In complex cyanotic heart disease, magnetic resonance imaging (MRI) and computed tomography (CT) are usually the imaging modalities of choice (Burchill et al. 2017). MRI excels at providing information on heart chamber volumines and can measure exact blood flows (Fratz et al. 2013). However, in patients with prosthesis and/or surgical metal clips, MRI images are often inconclusive due to extensive artifacts. Standard CT angiography will usually be used to demonstrate the heart and vascular anatomy and for planning operation strategy. Dynamic CT angiography can furthermore provide visual information on blood flow. Dynamic ^15^O-water positron emission tomography (PET) has previously been shown to be able to characterize the cardiopulmonary system in heart failure patients (Nielsen et al. 2019). The absence of metal artifacts in PET together with the ability of dynamic PET to track a bolus of ^15^O-water quantitatively in time through the cardiovascular system may thus complement information from MRI and CT.

## Case presentation

**Fig. 1:**
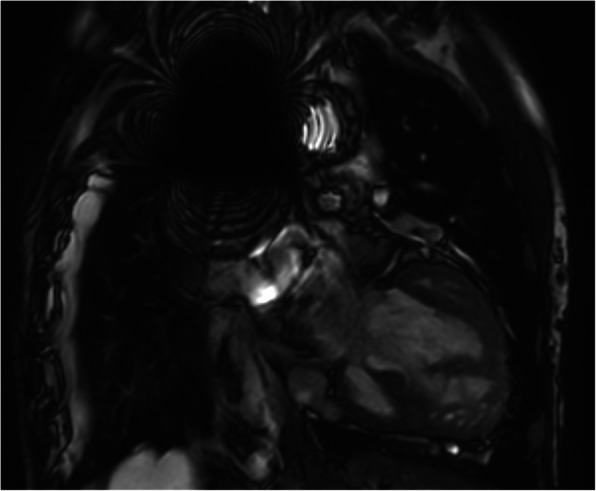
Sagittal steady-state free precession MRI image of the superior mediastinum. A large metal artifact caused by a surgical clip obscures the image and renders flow evaluation impossible

We present a case with a male born with univentricular heart (double inlet, double outlet left ventricle). Main pulmonary artery banding during childhood had failed to prevent pulmonary hypertension. Thus, establishment of Fontan circulation (connection of the superior and inferior vena cava to the pulmonary arteries) was contraindicated. Therefore, at 21 years of age, palliation of cyanosis was obtained with a bidirectional Glenn anastomosis (connection of the superior vena cava to right pulmonary artery). Due to shunting from the superior to the inferior vena cava, he developed increasing cyanosis which motivated closure of the azygos vein with a catheter-delivered metal coil. At 40 years of age, he developed signs of heart failure and had visible venous cutaneous collaterals. We examined his suitability for heart transplantation. We wanted to determine the pulmonary flow and vascular resistance, but measurement of the pulmonary blood flow is challenging in patients with complex cyanotic congenital heart disease with pulmonary blood supply from separate sources with different oxygen saturations. In these patients, standard invasive measurements using thermo dilution or Fick’s principle cannot be applied. In some patients, direct flow measurements in the pulmonary arteries can be done by phase-contrast MRI. However, many patients have metal implants that cause severe artifacts on MRI making flow measurements impossible. In this case, MRI flow measurements were attempted but were inconclusive due to artifact. Figure 1 shows an MRI scan of the superior mediastinum with profound artifact from a surgical metal clip. To examine the cardiopulmonary circulation and perfusion, a dynamic ^15^O-water PET scan was performed using bolus injection of ^15^O-water. Four hundred megabecquerels in 5 mL saline (1 mL s^−1^) was injected in a peripheral vein using an automatic injection system, followed by a 35-mL saline flush (2.0 mL s^−1^) as previously described (Nielsen et al. 2019). Together with the PET scan, a low-dose CT scan was performed for anatomical reference which allowed for the identification of pulmonary arteries, lung lobes, cardiac chambers, and the Glenn anastomosis. Regions of interest were drawn on the CT images and transferred to the dynamic PET.

**Fig. 2:**
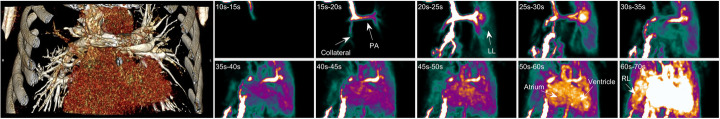
*Left*: Volume rendering of contrast CT of the superior mediastinum showing the pulmonary arteries. *Right*: Dynamic MIP PET images of ^15^O-water following a bolus injection. Collaterals are shown together with the pulmonary arteries (PA), the atrium, and ventricle. The left lung (LL) is perfused before the right lung (RL)

**Fig. 3:**
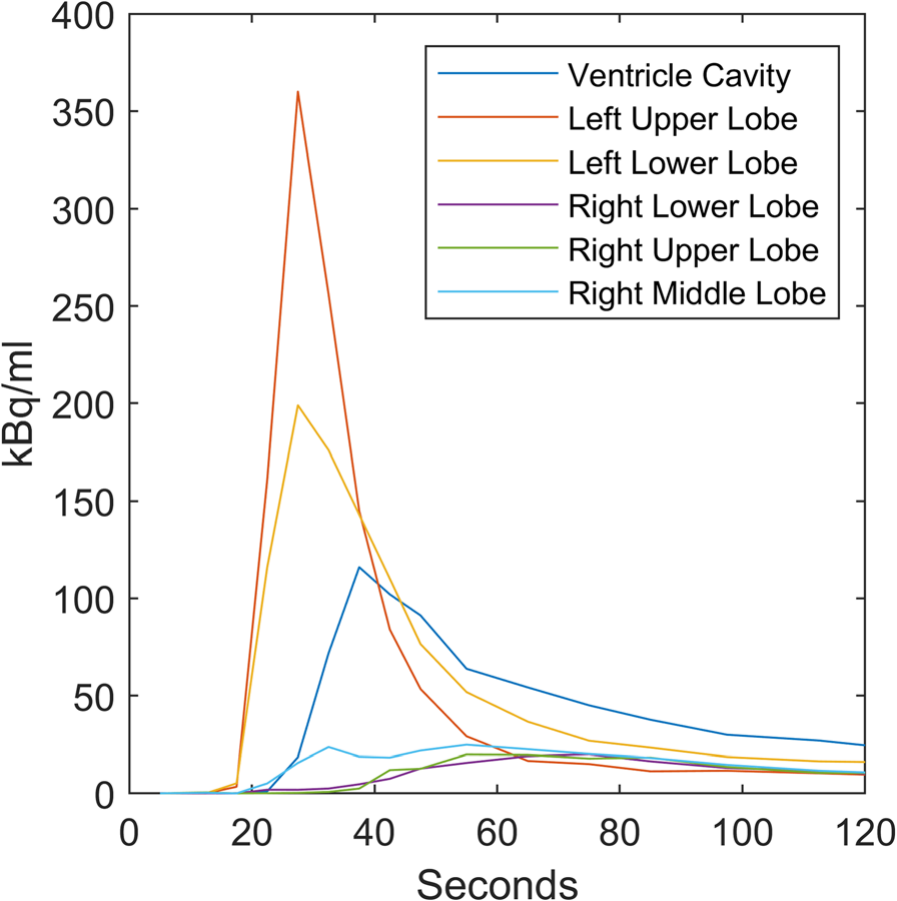
^15^O-water PET time-activity curves of the different lung lobes and the ventricle following a bolus injection at 0 s

**Fig. 4:**
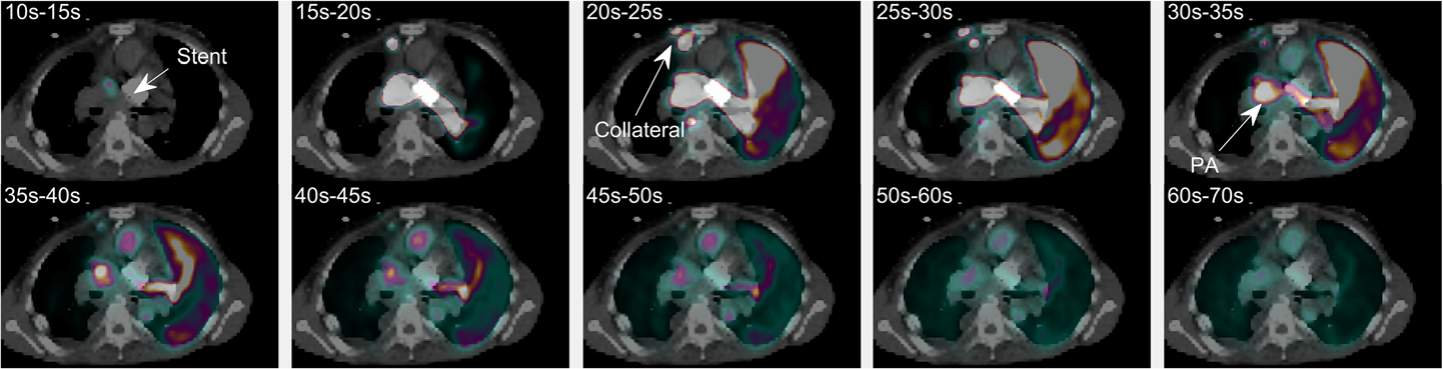
Transaxially fused images of dynamic ^15^O-water PET and CT. A pulmonary artery stent is shown together with collaterals and the pulmonary arteries (PA)

Figure 2 shows a dynamic CT angiography scan (left image) after a single contrast bolus and a ^15^O-water PET scan (right images) of the heart and lungs up to 70 s after bolus injection of ^15^O-water performed on separate dates. CT and PET scans are shown as maximum intensity projections (MIP). While the dynamic CT angiography scan showed that both lungs were indeed perfused, the ^15^O-water PET scan demonstrated that the blood from the superior vena cava primarily was led to the upper lobe of the left lung (Fig. 2, arrow—LL) which was the only lobe properly perfused. This is further illustrated by the time-activity curves of the lung lobes (Fig. 3). The time-activity curves show that a significantly lesser amount of blood was led to the left lower lobe compared to the left upper lobe and almost nothing reached the right lung. Moreover, the perfusion of the lobes of the right lung was delayed. Eventually, small quantities of blood reached the right lung approximately 30 s later than the left (Fig. 2, arrow—RL). This indicates that the blood from the superior vena cava reached the ventricle before it reached the right lung. Figure 4 shows fused images of the ^15^O-water PET scan and a low-dose CT scan performed simultaneously of the mediastinum and demonstrates the obtained PET information related to the anatomy of the mediastinum. The myocardial blood flow (MBF) of the ventricle was the same as a normal left ventricle (0.9 ml/ml/min). Time-activity curves are extracted from the major vessels and used for calculation of transit times and stroke volume using the distance between centroids and the area under the curve, respectively (Nielsen et al. 2019). The stroke volume index (SVI) in the aorta was low at 28 ml/m^2^, whereas 36 ml/m^2^ would be expected from a healthy person measured with this method. Furthermore, the mean transit time from the superior vena cava to the left ventricle was 28 s, which was approximately four times longer than would be expected in a healthy person. The scan demonstrated a comprehensive shunting of blood from the superior vena cava to the inferior vena cava through the internal thoracic vein and venous cutaneous collaterals (Fig. 2 and Fig. 4, arrow—collateral). Thus, approximately 35% of blood from the superior vena cava bypassed the lungs which was unknown before the PET scan.

^15^O-water is the gold standard for PET quantification of regional tissue perfusion. As a freely diffusible tracer, ^15^O-water has been intensively used to study the cardiopulmonary system including MBF and lung blood flow (Bergmann et al. 1984; Driessen et al. 2017; Harms et al. 2011; Heinonen et al. 2013). To our knowledge, this is the first report demonstrating that ^15^O-water PET can be used for evaluation of cardiopulmonary perfusion in complex congenital cyanotic heart disease.

## Discussion

^15^O-water PET scans provide dynamic images that easily track and quantify blood flow and perfusion, and the radiation dose of a single scan is very limited (0.4 mSv per injection). However, ^15^O-water PET scans cannot provide any substantial information on anatomy. Thus, this case demonstrates that multimodality imaging continuously is a necessity when assessing the circulation in congenital heart disease. Considering the additional information provided by a ^15^O-water PET scan in this case, we contemplate that the properties of the freely diffusible tracer ^15^O-water can be utilized in similar cases.

The patient was ultimately found unfit for cardiac transplantation due to significant comorbidities of kidney failure and liver disease secondary to his failing heart.

## Conclusion

This case demonstrates that the addition of a single ^15^O-water PET scan can provide extensive information on the cardiopulmonary circulation when examining complex cyanotic heart disease.

## Data Availability

Data sharing is not applicable to this article as no datasets were generated or analyzed during the current study.
